# The role of the type VI secretion system *vgrG* gene in the virulence and antimicrobial resistance of *Acinetobacter baumannii* ATCC 19606

**DOI:** 10.1371/journal.pone.0192288

**Published:** 2018-02-02

**Authors:** Jianfeng Wang, Zhihui Zhou, Fang He, Zhi Ruan, Yan Jiang, Xiaoting Hua, Yunsong Yu

**Affiliations:** 1 Department of Respiratory Diseases, The Affiliated Hospital of Hangzhou Normal University, Hangzhou, Zhejiang, China; 2 Department of Infectious Diseases, Sir Run Run Shaw Hospital, School of Medicine, Zhejiang University, Hangzhou, Zhejiang, China; 3 Key laboratory of microbial technology and bioinformatics of Zhejiang Province, Hangzhou, Zhejiang, China; nanyang technological university, SINGAPORE

## Abstract

The Type VI Secretion System (T6SS) is an important virulence system that exists in many bacterial pathogens, and has emerged as a potent mediator of pathogenicity in *Acinetobacter baumannii*. In this study, we inactivated one of the T6SS components *vgrG* (valine–glycine repeat G) gene in *A*. *baumannii* ATCC 19606 and constructed a complementation strain. BEAS-2b human alveolar epithelial cells was adopted to assess bacterial adhesion, and wild female BALB/c mice were used for *in vivo* experiments to assess the bacterial killing ability to host. Upon deletion of the *vgrG* gene, increased antimicrobial resistance to ampicillin/sulbactam, but reduced resistance to chloramphenicol were observed. The *vgrG* mutant strain showed lower growth rate, reduced eukaryotic cell adherence and impaired lethality in mice. However, the *vgrG* mutant strain is not implicated in biofilm formation. Our study suggests that the Type VI Secretion System core component VgrG contributes to both virulence and antimicrobial resistance in *A*. *baumannii* ATCC 19606.

## Introduction

*Acinetobacter baumannii* is one of the most common pathogens in hospital acquired infections, especially in patients with tracheal intubation or deep vein catheterisation, those who have undergone cerebral surgery, and those who are long-term bed ridden [1]. According to the 2014 CHINET Chinese bacterium antimicrobial resistance monitoring data, *Acinetobacter* spp. accounted for 11.11% of the total clinical isolates, and after *Escherichia coli* and *Klebsiella pneumoniae*, it is the third most commonly isolated Gram-negative bacteria [2]. Multi-drug resistance of *Acinetobacter baumannii* (MDR-Ab) has become a serious public health concern worldwide. The most notorious is carbapenem resistance, and resistance to colistin and tigecycline are also noteworthy [3]

The Type VI Secretion System (T6SS) is a phage-related system that exists in many bacterial pathogens, such as *Escherichia coli*, *Pseudomonas aeruginosa*, and *Burkholderia cenocepacia* [4–6]. T6SS is responsible for secretion of many toxic effector molecules that can kill both prokaryotic and eukaryotic prey cells [5–7]. The function of T6SS is similar to the tail of bacterial phage and consists of a floor structure corresponding to the cytosol dynamic organelles connected to the cytomembrane [7]. The valine glycine repeat G (VgrG) protein and hemolysin co-regulated protein (Hcp) tube complex translocate into the target cell via the dynamic activity of T6SS, the spike formed by the VgrG protein complex attacks the target cell by penetrating the membrane [8, 9]. Repizo et al. demonstrated that the Hcp secretion of T6SS influences the virulence of *A*. *baumannii* [10]. Weber BS et al. reported a multidrug resistance plasmid that contains a molecular switch for T6SS was identified in *A*. *baumannii* [11]. However, analysis of *A*. *baumannii* ATCC 19606 draft genome indicated that some genes located on plasmid in other strains are located within a larger genomic island that has been inserted into the chromosome of *A*. *baumannii* ATCC 19606 [12].

Various studies have revealed associations between resistance and virulence by *A*. *baumannii*. In particular, several regulatory genes, including RND-type efflux system, have been reported to be involved in the resistance and virulence of *A*. *baumannii* [13]. For example, the OmpA protein is involved in biofilm formation and virulence [14], it also can increase resistance to chloramphenicol, aztreonam, and nalidixic acid [15]. In this study, we investigated the role of VgrG in mediating *A*. *baumannii* ATCC 19606 resistance and virulence.

## Materials and methods

### Strains and plasmids

*A*. *baumannii* ATCC 19606 was used as the wild-type strain for all experiments. *A*. *baumannii* strains were grown on Mueller-Hinton (MH) or Luria Bertani (LB) agar at 37°C. Ticarcillin (TIC) at 100 μg/mL, kanamycin (KM) at 50 μg/mL, and tetracycline (Tc) at 10 μg/mL or 32 μg/mL were added for selection as needed [16]. The red fluorescent plasmid pNirFP-C was purchased from Haimaiyueer Biotechnology Limited (Hangzhou, China). The plasmids pAT03 (pMMB67EH with FLP recombinase fragment) and pAT04 (pMMB67EH with RecAb system) were donated by Bryan Davies (University of Texas at Austin, Texas, USA). The strains *Salmonella Typhimurium* XH118 carrying a blue fluorescent gene and *Salmonella Typhimurium* XH119 carrying a green fluorescent gene were donated by Dan Andersson from Uppsala University in Sweden.

### *vgrG* knockout mutant

In-frame gene deletions were constructed as previously described, with modifications [16]. The *vgrG* gene was selected from the *A*. *baumannii* ATCC 19606 genome for targeted deletion: DJ41_2425 (from 3454756 to 3457122) (S1 Table). Briefly, a kanamycin resistance cassette and 800 to 1,200 bp fragments corresponding to the regions up and downstream of the respective genes were cloned into pCR2.1-TOPO (Invitrogen). Subsequently, the homologous fragment was electroporated into *A*. *baumannii* carrying RecAb on pMMB67EH (pAT04 plasmid), which was maintained with carbenicillin. The kanamycin cassette in successful recombinants was deleted by pMMB67EH expressing the FLP recombinase (pAT03 plasmid). Polymerase chain reaction (PCR) and sequencing were used to confirm the loss of the kanamycin cassette and the plasmids pAT04 and pAT03. Complementation using pWH1266 was performed as described elsewhere to generate pWH1266-Δ*vgrG* [17]. The fluorescence genes were also integrated into the chromosome by homologous recombination.

The promoter sequence of fluorescent gene was amplified from *A*. *baumannii* ATCC17978 (gene number: A1S_2840). The blue and green fluorescent gene sequence were amplified separately from *Salmonella Typhimurium* XH119 and *Salmonella Typhimurium* XH119 (S1 Table). After the target gene fragments were purified with gel extraction method, they were linked with the linearized plasmid pWH1266 by the recombinant enzyme. After the pWH1266 plasmid with the green fluorescent gene was extracted, it was transformed into *A*. *baumannii* ATCC19606 by electroporation and constructed the wild green fluorescen strain. The blue fluorescence gene with promoter was amplified from the modified plasmid pWH1266 with blue fluorescence, and replaced the *vgrG* gene (DJ41_2425) in *A*. *baumannii* ATCC19606 by homologous recombination. The BEAS-2b human alveolar epithelial cells were transfected with the red fluorescent plasmid pNirFP-C.

### Antimicrobial susceptibility assays

Antimicrobial susceptibility testing for amoxicillin/clavulanic acid, chloramphenicol, piperacillin, ampicillin/sulbactam, tetracycline, minocycline, colistin, ciprofloxacin, meropenem, and amikacin was performed using E-test strips for three replicates. *E*. *coli* ATCC 25922 was used as the quality control strain. For the susceptibility test, a single colony was cultured at 37°C overnight on a Mueller-Hinton (MH) agar plate, recovered and diluted to 0.5 McFarland turbidity in a 0.9% NaCl (w/v) solution. The diluted suspension was the spread on an MH plate with a cotton swab. After the antimicrobial susceptibility test strips were applied, the plate was incubated at 37°C for 22 h. The data interpretation was performed in accordance with the Clinical and Laboratory Standards Institute (CLSI) 2016 guidelines.

### Growth and biofilm assays

Bacterial cells were dispensed into a 96-well microtiter plate at an OD_600_ of 0.01 in 100 μL of the growth media and were grown at 37°C with shaking for 16 h [18]. Growth was measured every 5 min for 16 h by Spectrum Microplate Spectrophotometer (OD_600_); data represent the average of three independent experiments ± standard deviation. The growth rate was estimated based on the OD_600_ curves using R script [19].

Biofilm formation was assessed as described previously[20]. Biofilm formation on the bottom of a microtiter dish was assessed by crystal violet staining of cells statically cultured in M63 as described previously [20]. Biofilms were colorimetrically quantified at A550 nm. For quantitative assays, the assays were performed in four biological replicates and repeated at least twice using fresh biological samples each time.

### BEAS-2b adhesion assays

To assess bacterial adhesion, BEAS-2b human alveolar epithelial cells were cultured in 6-well plates in 5% CO_2_ at 37°C with 15% heat-inactivated foetal bovine serum [21, 22]. Microscope slides were placed in the bottoms of the 6 wells plate prior to the addition of 1 ×10^5^ cells per well. After culturing the cells for 24 hours, the red fluorescent plasmid pNirFP-C was added, and the cells were cultured for 24 additional hours before the bacteria were added. Single colonies of *A*. *baumannii* were cultured in LB with shaking overnight at 37°C and then subcultured 1:100 to fresh LB and incubated with shaking for 3 h. The bacteria were washed three times with pre-warmed PBS solution and quantified by optical density. Then, 1×10^7^ bacterial cells were added to each well. The bacteria were incubated with the BEAS-2b cells for 2 hours at 37°C, the confluent monolayer cells were washed three times with pre-warmed PBS solution, and the microscopy slides were removed and viewed under the fluorescent microscope. At the same time, the replicate wells were treated with a 0.25% trypsin solution containing 0.02% EDTA, and colony-forming units (CFUs) were quantified by plating serial dilutions on MH agar. For microscopy purposes, the cells were transfected with red fluorescent plasmid pNirFP-C, the wild bacterial strain were transformed with green fluorescent plasmid, and the mutant strain had a blue fluorescence gene integrated into the chromosome. All bacterial adhesion assays were run as three biological replicates.

### Mouse bacteremia model

Single colonies of *A*. *baumannii* were cultured in LB with shaking overnight at 37°C, subcultured 1:100 in fresh LB, and adjusted to a density of 0.1 at OD_600_. After the bacteria were cultured in LB for 3 h, they were washed 3 times in phosphate-buffered saline (PBS) and quantified by optical density (approximately 5×10^8^ CFUs/ml) for injection or *in vitro* assay. A total of 15 wild female BALB/c mice were used for all *in vivo* experiments and repeated twice. Mice were injected intravenously with 5×10^7^CFUs inoculum of *A*. *baumannii* in 100 μL of sterile PBS. The mice were monitored for survival for longer than 72 h. CFUs were quantified by serial dilutions on MH agar. All animal treatments were carried out in accordance with the National Institutes of Health Guide for the Care and Use of Laboratory Animals and approved by the Institutional Animal Care and Use Committee of Zhejiang University.

### Statistical analysis

The levels association of growth rate, biofilm and cell adhesion between *A*. *baumannii* ATCC 19606 and the mutant strains were analysed using the SPSS Statistics 19.0 software. According to the normality test and homogeneity of variances test, the levels of growth rate, biofilm and cell adhesion appear to be a normal distribution with equal variance, so all the data was adopted with analysis of variance (ANOVA) statistical test. The survival rate of mice was compared by Chi-Squared Test (Fisher probabilistic method). Statistical significance was established by using a conventional level of *P* < 0.05.

## Results

### Effect of *vgrG* mutations on bacterial resistance

To study the virulence and antimicrobial role of *vgrG*, we constructed mutant strains in *A*. *baumannii* ATCC 19606 (Table 1). We also compared the antimicrobial susceptibility between the wild-type strain and the mutant strain. Compared with the wild-type strain, the MIC of the mutant strain ATCC 19606Δ*vgrG* to ampicillin/sulbactam was increased (2 to 32 μg/mL), but the reduced resistance to chloramphenicol was also detected (>256 to 96 μg/mL) (Table 2). When the *vgrG* gene was complemented, the antimicrobial sensitivity of the complementary strain *A*. *baumannii* ATCC 19606*ΔvgrG*-C was recovered to the level of the wild-type strain. The antimicrobial susceptibility assays showed that the strain containing the blue fluorescence gene in place of *vgrG* was consistent with the gene knockout strain of *vgrG*, demonstrating that the genetic manipulation did not affect the antimicrobial susceptibility of these strain. Our data also demonstrated that *vgrG* gene has no effect on susceptibility to amoxicillin/clavulanic acid, piperacillin, tetracycline, minocycline, colistin, ciprofloxacin, meropenem, and amikacin in *A*. *baumannii* ATCC 19606.

**Table 1:** Strains used in this study.

Strain	Annotation
ATCC 19606	ATCC 19606 wild-type strain
ATCC 19606*ΔvgrG*	ATCC 19606 *vgrG* knockout strain
ATCC 19606*ΔvgrG*::*BFP*	ATCC 19606 *BFP* replaced *vgrG*
ATCC 19606*ΔvgrG-*C	ATCC 19606 *vgrG* complementary strain
ATCC 19606*ΔvgrG*::*BFP*-C	ATCC 19606 *BFP vgrG* complementary strain

**Table 2:** Effect of *vgrG* on MICs of antimicrobials in *A*. *baumannii* ATCC 19606.

Antimicrobial agents	MIC (μg/mL)				
	ATCC 19606	ATCC 19606*ΔvgrG*	ATCC 19606*ΔvgrG*::*BFP*	ATCC 19606*ΔvgrG*-C	ATCC 19606*ΔvgrG*::*BFP* -C
XL	>256	>256	>256	>256	>256
CL	>256	96	128	>256	>256
PP	96	256	256	128	128
AB1/2*	2	32	32	2	2
TC	3	2	2	2	2
MC	0.125	0.125	0.125	0.25	0.38
CO	0.125	0.125	0.125	0.125	0.125
CI	0.38	0.38	0.38	0.38	0.38
MP	0.75	0.75	0.75	0.75	0.75
AK	3	3	4	3	4

Note: *XL* amoxicillin/clavulanic acid; *CL* chloramphenicol; *PP* piperacillin; *AB1/2* ampicillin/sulbactam; *TC* tetracycline; *MC* minocycline; *CO* colistin; *CI* ciprofloxacin; *MP* meropenem; *AK* amikacin.

*The categories of susceptibility to ampicillin/sulbatam was changed from susceptible to resistance in the mutant strain ATCC 19606Δ*vgrG*.

### Effect of *vgrG* mutations on bacterial growth rates

**Fig 1:**
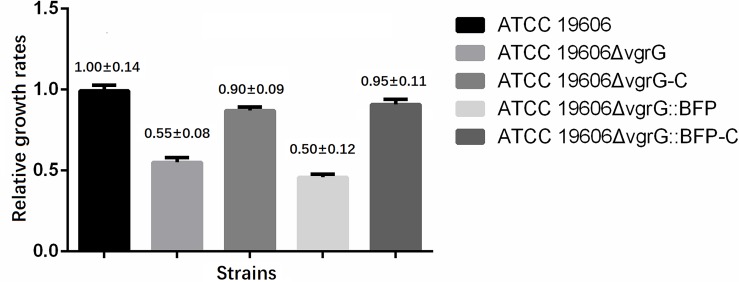
Effect of *vgrG* mutations on bacterial growth rates. Bacterial cells were dispensed into a 96-well microtiter plate and incubated at 37°C with shaking for 24 h. The growth rate of the mutant strain (*A*. *baumannii* ATCC 19606Δ*vgrG*) decreased remarkably compared to the parental strain.

Compared to the wild-type strain *A*. *baumannii* ATCC 19606 (1.00±0.14), the growth rate of the mutant strain ATCC 19606*ΔvgrG* (0.55±0.08) was significantly decreased (*P* < 0.01) (Fig 1). After the *vgrG* gene was complemented (ATCC 19606*ΔvgrG*-C), the growth rate was not completely recovered (0.90±0.09), which was possibly due to the plasmid-mediated growth perturbation. These data suggested that the expression of *vgrG* gene contributes to bacterial growth. The growth rates variation of the two mutant strains ATCC 19606*ΔvgrG*::*BFP* (0.50±0.12) and ATCC 19606*ΔvgrG*::*BFP*-C (0.95±0.11) were congruent with that of ATCC 19606*ΔvgrG* and ATCC 19606*ΔvgrG*-C, respectively.

### Effect of *vgrG* mutations on bacterial biofilm formation

Compared with the wild-type strain *A*. *baumannii* ATCC 19606 (OD_550_ = 0.79±0.12), the biofilm formation of the mutant strain *A*. *baumannii* ATCC 19606*ΔvgrG* (OD_550_ = 0.73±0.05) was similar (*P* > 0.05). These results indicated that the *vgrG* gene was not implicated in *A*. *baumannii* biofilm formation.

### Effect of *vgrG* mutations on *A*. *baumannii* adhesion to lung epithelial cells

Wild-type and mutant strains of *A*. *baumannii* were added to the cell culture dish and co-cultured with BEAS-2b human alveolar epithelial cells for 2 hours. Following this co-culture period, the numbers of bacteria adhering to the BEAS-2b human alveolar epithelial cells per well were determined. As a result, the numbers of the wild-type strain ATCC 19606 was (2.6±0.6)×10^5^ CFUs, while the numbers of the mutant strain ATCC 19606Δ*vgrG* was (1.1±0.9)×10^4^ CFUs, and ATCC 19606*ΔvgrG*-C was (1.2±0.7)×10^5^ CFUs. *A*. *baumannii* ATCC 19606 cell counts were ten-fold higher than the *ΔvgrG* mutant strain (*P* < 0.01).

**Fig 2:**
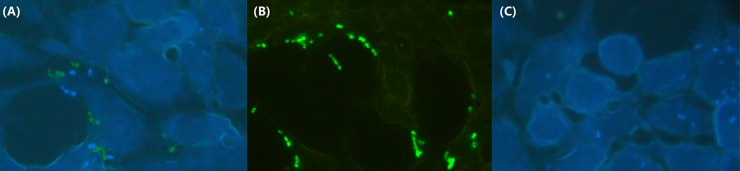
*A*. *baumannii* ATCC 19606 adhesion to lung epithelial cells. BEAS-2b lung epithelial cells were transfected a plasmid encoding red fluorescent protein, and 1×10^7^ bacterial per well were added. The cells were co-cultured for 2 hours in 5% CO_2_ at 37°C with 15% heat-inactivated foetal bovine serum. Under the same field of view, the wild-type strain, which were transformed with a plasmid encoding green fluorescent protein, were more concentrated than the mutant strain, which contained a gene encoding blue fluorescent protein integrated into the chromosome. Note: (A) *A*. *baumannii* ATCC 19606 wild and mutant strains; (B) *A*. *baumannii* ATCC 19606 wild-type strain; (C) *A*. *baumannii* ATCC 19606 mutant strain.

When the co-culture system of *A*. *baumannii* and BEAS-2b cells was observed under fluorescence microscopy, we found that *A*. *baumannii* ATCC 19606 adhered to the lung epithelial cells (Fig 2). Under the same field of view, the wild-type strain that were transformed with a green fluorescent plasmid were more concentrated than the mutant strain that contained a blue fluorescence gene integrated into the chromosome, suggesting that the cell adhesion ability of the wild cells was stronger than that of the knockout strain under the same conditions. The results demonstrated that the *vgrG* gene was beneficial to bacterial cellular adherence.

### Virulence in mouse model of systemic infection

**Fig 3:**
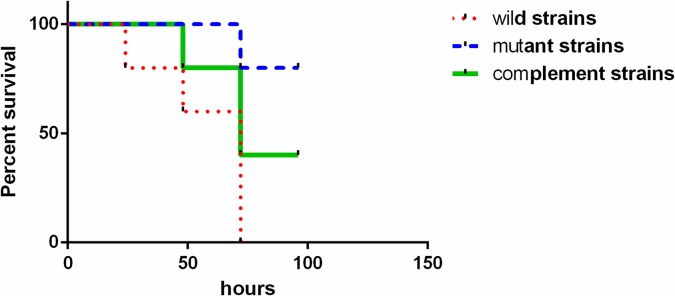
Effect of *vgrG* on survival of mice infected with *A*. *baumannii* ATCC 19606. Mice were injected with wild *A*. *baumannii* ATCC 19606 at a dose of 5×10^7^ CFUs in the tail vein. All mice died after 72 hours. When the mice were infected with the mutant strain, only one mouse died after 72 hours.

When the mice were injected with (4.9±0.8)×10^7^ CFUs of the wild-type strain *A*. *baumannii* ATCC 19606 through the tail vein, all mice died after 72 hours (Fig 3). However, when the mice were injected with (5.1±0.7)×10^7^ CFUs of the strain *A*. *baumannii* ATCC 19606Δ*vgrG* through the tail vein, their physical performance declined after 72 hours, but only one fifth mice died. When the mice were injected with (5.0±0.9)×10^7^ CFUs of the *vgrG* complementary strain, three fifth mice died after 72 hours. The results confirmed that the virulence of *A*. *baumannii* ATCC 19606 declined when *vgrG* was deleted. Thus, *vgrG* played an important role in enhancing the virulence of *A*. *baumannii* ATCC 19606. The lethal ability of wild-type *A*. *baumannii* ATCC 19606 to mice was stronger than that of the *vgrG* mutant strain (*P* < 0.05).

## Discussion

**In recent years, T6SS has increasingly attracted attention because it is present in** many bacterial pathogens. By delivering virulence effector proteins into target cells, T6SS plays an important role in bacterial virulence [7, 23]. Comparative genomics suggests that T6SS is present in most *A*. *baumannii* strains [24]. Although T6SS plays an important role in the virulence of *A*. *baumannii* but has no effect on biofilm formation [10].

In this study, we inactivated the *vgrG* gene, which is a core element of *A*. *baumannii* T6SS. Our experimental results showed that murine bloodstream mortality caused by an *A*. *baumannii* ATCC 19606 *vgrG* mutant strain was lower than that of the wild-type strain, which confirmed that *vgrG* plays an important role in *A*. *baumannii* virulence. We also compared the biofilm formation ability between the wild-type strain ATCC 19606 and their *vgrG*-knockout mutant strain. The results confirmed *vgrG* was not implicated in biofilm formation in *A*. *baumannii*. Thus, the contribution of *vgrG* to virulence might be due to other mechanisms.

Bacterial virulence factors can affect bacterial pathogenesis by influencing the bacterial growth, aggregation, adhesion, invasion and toxin secretion. For *A*. *baumannii*, adhesion ability is an important indicator of its virulence. Due to its powerful adhesion ability, *A*. *baumannii* often causes persistent infections [25]. The ability of *Escherichia coli* to adhere to lung epithelial cells decreased when the expression of T6SS was down regulated. Desilets M et al. isolated mucosa-associated *E*. *coli* from patients with inflammatory bowel disease (IBD), including 14 strains which were considered as adherent-invasive *Escherichia coli* (AIEC) and were acquired from IBD patients by intestinal biopsy. Comparative genomics analysis suggested the bacterial adhesion and invasion ability of *E*. *coli* was highly correlated with T6SS [26]. There were lack of research data focused on the specific role of T6SS in *A*. *baumannii* adhesion and invasion, and more attention has been paid to the role of T6SS in the competition between *A*. *baumannii* and other bacteria [8, 27]. Our study showed that the growth rate of *A*. *baumannii* was reduced when *vgrG* was deleted and the adhesion ability of *A*. *baumannii* ATCC 19606 mutant strain also decreased compared to that of wild-type strain. Our study therefore proved that *vgrG* played an important role in *A*. *baumannii* adhesion.

Previous studies of T6SS have investigated its virulence potentials, and it was not known whether T6SS altered *A*. *baumannii* antimicrobial resistance. Our study showed that the mutant strain had increased antimicrobial resistance to ampicillin/sulbactam, but reduced resistance to chloramphenicol. The mechanisms of resistance to chloramphenicol and β-lactam antibiotics in *A*. *baumannii* are completely different; chloramphenicol resistance is often due to the efflux pumps [28, 29], while oxacillinases are the major contributors to carbapenem resistance [30]. It is reasonable to postulate that T6SS could become a therapeutic target allowing to circumvent certain existing antibiotic resistance.

In summary, when the *vgrG* gene of *A*. *baumannii* was deleted, the growth rate was reduced, the adhesive and invasive ability declined, and murine mortality was decreased, demonstrating that *vgrG* plays an important role in the virulence of *A*. *baumannii*. The *vgrG* gene can also increase antimicrobial resistance to ampicillin/sulbactam, but reduce resistance to chloramphenicol. Our study suggests the T6SS *vgrG* gene of *A*. *baumannii* ATCC 19606 has a dual role in virulence and antimicrobial resistance, and further studies in this field are warranted.

## Supporting information

S1 Table *A. baumanni* ATCC 19606 genetic manipulation PCR primers. (DOC)
