# Supplementary material for: The role of the type VI secretion system vgrG gene in the virulence and antimicrobial resistance of Acinetobacter baumannii ATCC 19606
Source: PLoS One. 2018 Feb 2;13(2):e0192288. doi: 10.1371/journal.pone.0192288 (PMC5796710; doi:10.1371/journal.pone.0192288)
Supplement: S1 Table — (DOC) [file pone.0192288.s001.doc]

**Table S1** *A. baumanni* ATCC 19606 genetic manipulation PCR primers.

| Primers | Sequence |
| --- | --- |
| 19606*vgrG*-TF | 5'-TCTAGAGGATCTACTAGTCATATGGAT CAACGAACAGGAGCATCAG-3' |
| 19606*vgrG*-P2 | 5'- GAACTTCGAAGCAGCTCCA GCCCTCATTAAGATAATGCTG-3' |
| 19606*vgrG*-P3 | 5'-AGGAACTAAGGAGGATATTCATATGTTAAGTTCTGCGATAGTTTGCC-3' |
| 19606*vgrG*-TR | 5'-TTCGAGCTCGGTACCCGGGGATCCGATCGATGACAGGTGATAATTCAGA-3' |
| 19606*vgrG*-KanaF | 5'-GTTCGATTATGATCTAAATTTATAAAAATGTATGGAGCTGCTTCGAAGTTC-3' |
| 19606*vgrG*-KanaR | 5'-GGCAAACTATCGCAGAACTTAACATATGAATATCCTCCTTAGTTCCT-3' |
| 19606vgr-1266PstI-F | 5'-TTGCGCAACGTTGTTGCCATTGCTGCAATGACAGCCGAGCTGATTTG-3' |
| 19606vgr-1266PvuI-R | 5'-ACTGCGGCCAACTTACTTCTGACAACGCACCTCACCAACCTCTCAA-3' |
| promoter-F | 5'-CGGGATCCCGTGGCTATAACTGACATAATCACAAG-3' |
| promoter-R | 5'-TAGTATTTCTCCTCTTTCTCTGAATAGTAGCAAGTGCAATACGACTCAA-3' |
| GFP/BFP-F | 5'-TTGAGTCGTATTGCACTTGCTACTATTCAGAGAAAGAGGAGAAATACTA-3' |
| GFP/RFP-R | 5'-CCGCTCGAGCGGGTTCACCGACAAACAACAGATA-3' |
